# Biomarkers in Neurodegenerative Diseases

**DOI:** 10.3390/biomedicines10020215

**Published:** 2022-01-20

**Authors:** Arnab Ghosh

**Affiliations:** Center for Gene Regulation in Health and Disease, Department of Biological, Geological and Environmental Sciences, Cleveland State University, Cleveland, OH 44115, USA; a.ghosh100@csuohio.edu

An increasing number of people are affected by various neurodegenerative diseases each year, impacting the quality of life of millions of people worldwide. However, considerable knowledge gaps in the mechanistic understanding of these diseases present a challenge to address this threat to human life successfully. Recent endeavors in basic research, clinical trials, and other research areas have provided vital insights to discover novel biomarkers for improved diagnosis and better treatment options for patients suffering from such diseases. Articles published as part of the Special Issue “Biomarkers in Neurodegenerative Diseases” highlight the recent advances in this field and emerging strategies to counter such diseases in the future.

Three research articles published in the Special Issue focused on molecular mechanisms involving Alzheimer’s disease (AD). First, Paik et al. used human SH-SY5Y neuroblastoma cells to show the neuroprotective role of Somatostatin-14 (SST). The possible mechanism involves the ability of SST to regulate the intracellular calcium levels and Collapsin Response Mediator Protein 2 (CRMP2) phosphorylation [1]. Second, Akyuz and coworkers measured the expression of neuronal inwardly rectifying potassium (Kir) channels in an Aβ(1–42)-infused rat model of AD [2]. These findings open up newer possibilities to discover novel biomarkers or therapeutic targets against AD in the future. Finally, Ahmad et al. showed that lupeol (a plant-based triterpenoid compound) could improve memory function in the AD mouse model. Lupeol’s antioxidant and anti-inflammatory properties were proposed to be responsible for this observation [3]. Naturally occurring biologically active compounds present a promising target to evaluate as a potential therapy against many pathological conditions, including neurodegenerative diseases.

Kessler and others show a correlation between dysregulation of Monopolar Spindle 1 Kinase (MPS1) and tumor aggressiveness in glioblastoma multiforme (GBM) patients [4]. The researchers performed qPCR on frozen tumor tissue samples of GBM patients and compared them with non-pathological tissue samples. However, a more extensive study involving an increased number of patients is necessary to test whether MPS1 mRNA levels can be a reliable indicator of tumor aggressiveness in cancer patients across different subgroups.

One key aspect of neurologic disorders includes complications arising from traumatic brain injuries (TBIs). In this regard, Alam et al. investigated the role of quinpirole in dopamine D2 receptor (D2R) activation using a mouse model simulating TBI [5]. This study implicated quinpirole in D2R regulation and suggested a mechanism via the Akt/GSK3-β signaling pathway, leading to brain function recovery by reducing inflammatory responses. This observation opens up additional prospects to improve brain functions in patients suffering neurologic ailments due to TBI. Nevertheless, further studies are necessary to test the hypothesis definitively.

Fulgenzi et al. provide an excellent update in the investigation of chelation therapy involving EDTA as a potential treatment for neurologic disorders. They studied more than 300 patients from a broad age group to monitor their toxic metal burdens before and after receiving EDTA as part of the therapy [6]. Information obtained from the study provides excellent guidance for more detailed analyses involving larger patient groups to investigate the efficacy of chelation therapy in the future.

One of the critical challenges for better treatment outcomes is correctly identifying the causes behind indicators of different neurologic conditions. Chien and coworkers used an artificial neural network to distinguish between dopamine transporter single-photon emission computed tomography (DAT-SPECT) images obtained from patients with Parkinson’s disease or parkinsonism resulting from other illnesses with high (more than 80%) accuracy [7].

Apart from these original research papers, the Special Issue also contains an excellent collection of review articles highlighting recent progress, challenges, and future directions in the field. For example, the review articles published by Jacopo Meldolesi and Bălașa et al. are excellent resources summarizing the significant advances made in fluid and imaging biomarkers for the early detection of neurodegenerative disorders in recent times [8,9]. The authors also make a great effort to outline the deficiencies of our present knowledge in the field and potential ways to address these shortcomings. Giorgi et al. focused on recent advancements in understanding the mechanisms of autophagy and mitophagy processes and their relevance to neurodegenerative diseases [10]. Knowledge obtained from studying these cellular processes could lead to better therapeutic strategies against such conditions in the future. Finally, Bordoni and colleagues summarized the significance of dysregulation of a transcription factor called Nuclear Receptor Related 1 (NURR1) protein for perinatal stress [11]. Such stress is known to substantially increase the possibility of chronic diseases (such as neurologic conditions) later in life.

I hope that these excellent research works and review articles published as part of the Special Issue will provide an invaluable reference for further research to counter the menaces posed by neurologic disorders in the future.

## Data Availability

Not applicable.
